# Is the In Vitro Observed NETosis the Favored Physiological Death of Neutrophils or Mainly Induced by an Isolation Bias?

**DOI:** 10.3390/ijms24087368

**Published:** 2023-04-17

**Authors:** Julia Rimboeck, Michael Gruber, Sigrid Wittmann

**Affiliations:** Department of Anaesthesiology, University Hospital Regensburg, 93042 Regensburg, Germany

**Keywords:** neutrophils, PMNs, isolation, apoptosis, NETosis, lifespan, neutrophil functions, *g*-time

## Abstract

Centrifugation steps are regularly used for neutrophil isolation. Thereby, the influences of applied *g*-forces on the functionality of PMNs have hardly been analyzed and could consequently have been overlooked or led to biased results. We now hypothesize that blood PMNs—when gently isolated—can be long-lived cells and they physiologically become apoptotic rather than NETotic. Neutrophils were isolated from whole blood without centrifugation using a sedimentation enhancer (gelafundin). PMNs were analyzed via live-cell imaging for migratory activity and vitality condition by fluorescent staining. Native neutrophils showed still relevant migratory activity after more than 6 days ex vivo. The percentage of cells that were annexin V^+^ or PI^+^ increased successively with increasing ex vivo time. In addition, the characteristics of DAPI staining of gently isolated granulocytes differed markedly from those obtained by density gradient separation (DGS). We conclude that NETosis occurring after DGS is the consequence of applied *g*-forces and not a physiological phenomenon. Future studies on neutrophils should be performed with most native cells (applied *g*-time load as low as possible).

## 1. Introduction

Neutrophils (polymorphonuclear leukocytes; PMNs) make up the largest proportion of leukocytes in the human blood and form an essential part of the innate (nonspecific) immune system [1]. However, when misguided or malfunctional, the cellular innate immune response may also be pathological: PMNs are thought to play an important role in the pathogenesis of several diseases, such as sepsis [2], systemic lupus erythematosus [3], rheumatoid arthritis [4] or cystic fibrosis [5]. 

Neutrophils have been considered short-lived cells, circulating half-time in blood less than 24 h and being gradually cleared in various tissues [6,7]. 

There is some discussion about the possibility of a longer lifespan of neutrophils in peripheral blood under physiological conditions: Pillay et al. reported a median lifespan of 5.4 days [8], but this has been questioned [9,10]. However, a definitive clarification of the issue could not be achieved so far. 

It has been described that there are different possible modes of death of neutrophils, including NETosis and apoptosis [11,12]. 

The formation of extracellular traps (NETs) is considered the final and one of the most important ways for PMNs to defend the organism against pathogens [13]. This process is promoted by reactive oxygen species (ROS) leading to a violent disruption of the nuclear and cellular membrane and release of chromatin, DNA, citrullinated histones and contents of intracellular granules [14]. In previous publications of our laboratory, by default, NETosis was observed within 8 h when PMNs were isolated by density gradient separation (DGS) [15,16,17]. 

In contrast to NETosis, during apoptosis, different signal characteristics, e.g., the condensation of genetic material, the loss of plasma membrane asymmetry leading to extracellular exposure of phosphatidylserine (PS) and the activation of caspases, can be observed [11,18]. 

Annexin V-FITC can bind to PS and, therefore, can be used as an apoptosis marker [19]. Propidium iodide (PI) is a DNA dye that can be used to detect non-vital cells because it cannot passively traverse into cells which have an intact plasma membrane [20]. Differentiation between vital, apoptotic and dead cells can be made by simultaneous use of both dyes and fluorescence analysis: vital cells are both annexin V^−^ and PI^−^; (early) apoptotic cells are annexin V^+^ but PI^−^; (late) apoptotic/necrotic cells are both annexin V^+^ and PI^+^; necrotic/dead PMNs are annexin V^−^ and PI^+^. This classification of the vitality state of cells was mainly applied by flow cytometry [21,22,23].

To better understand the physiological and pathological behavior of neutrophils, it is inevitable to use most native PMNs to represent the in vivo situation as accurately as possible. 

Standard procedures to isolate neutrophils are, for instance, the density gradient separation [15,16,17], magnetic-activated [24] or fluorescence-activated cell sorting [25]. These methods have in common that they involve centrifugation steps. Consequently, the cells are subjected to *g*-forces during the isolation process. However, as recently found, centrifugation implements a dramatic paralytic inhibition of PMN functions beyond a certain *g*-time (defined as product of centrifugation duration and applied *g*-force) [26]. 

To obtain PMNs as native as possible, we used a gentle isolation method without centrifugation using gelafundin as sedimentation enhancer. To test our hypothesis, that the exposure to *g*-times during cell isolation process (here: DGS) is responsible for the sudden NETotic death of PMNs in vitro, we aim to answer the following questions in this study:

Are gently isolated (non-centrifuged) blood PMNs still short-lived cells under in vitro observation? How do non-activated neutrophils die, if not in NETosis? How long post withdrawal can relevant migratory activity be detected and how does it change with increasing observation time? 

## 2. Results

Whole blood was collected from a total of six healthy donors who had the following characteristics (Table 1). 

### 2.1. Chemotactic Migration

The experimental setup was tested in preliminary trials (n = 6). In these, the time required for PMNs to migrate from the reservoirs into the gel matrix of the observation channels was investigated. PMNs arrived in the observable part of the channels after a median of 17.2 h. Based on this result, the ongoing migration evaluation started 24 h post withdrawal. 

To evaluate the migration of PMNs, we compared 15-min observation periods 24–150 h post withdrawal. Only cell tracks that could be observed for at least 450 s and had a track length (TL) ≥ 25 µm were considered for further analyses. 

#### 2.1.1. Effect of Increasing Ex Vivo Time on Neutrophil TL and TS

**Figure 1:**
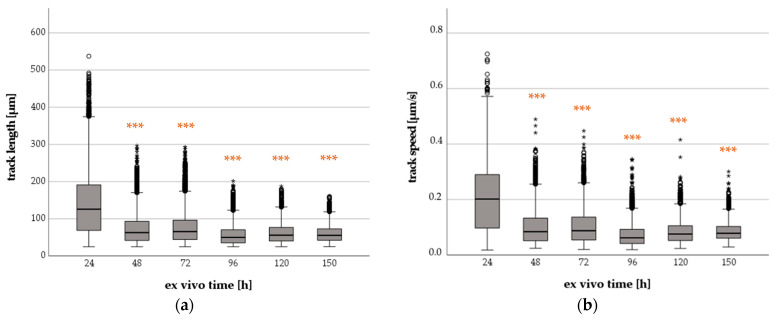
Progress of median TL (**a**) and median TS (**b**) of PMNs with increasing ex vivo time. Significant differences between groups compared with ex vivo time of 24 h are orange colored (*** *p* < 0.001), small circles in graphics mark statistical outliers (≥1.5 × IQR) and asterisks display extreme values (≥3 × IQR).

Firstly, we investigated the migration of PMNs in terms of TL and mean track speed (TS) after a gentle cell isolation without centrifugation steps (Figure 1).

The PMNs showed migratory activity until up to 150 h ex vivo. The median TL [µm] and the median TS [µm/s] decreased significantly when comparing 24-h (TL: 126; TS: 0.20) and 48-h (TL: 63; TS: 0.10) values. The medians remained almost unchanged after 72 h (TL: 66; TS: 0.10), 96 h (TL: 50; TS: 0.07), 120 h (TL: 56; TS: 0.08) and 150 h (TL: 56; TS: 0.08). TL and TS values were significantly higher after 24 h (*p* < 0.001) compared to all subsequent observation periods.

#### 2.1.2. Effect of Increasing Ex Vivo Time on Analyzed PMN Numbers

**Figure 2:**
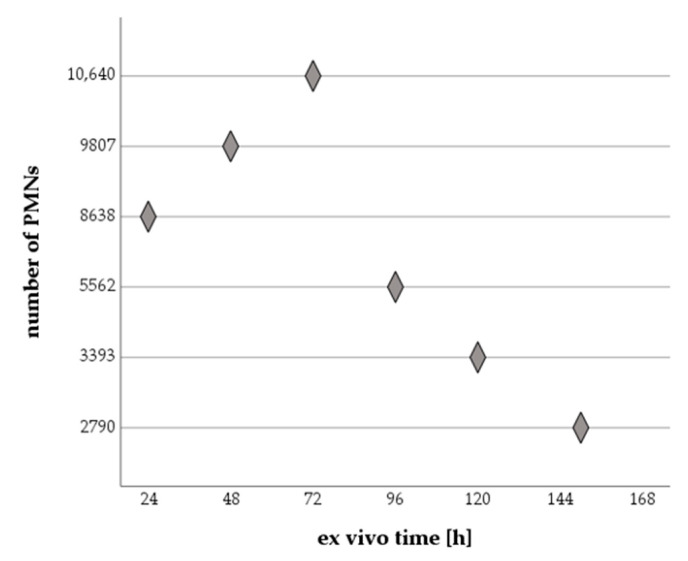
Total number of PMNs analyzed with increasing ex vivo time.

All PMNs that met the above criteria were included in the migration analyses (Figure 2). 

The absolute number of cells in the observation area increased continuously after 24 h and reached its maximum after 72 h. During this phase, cells migrated steadily from the reservoirs into the channel matrix. The number of newly migrated cells exceeded the number of PMNs that stopped migrating. Over the next 24 h (ex vivo time: 72 h to 96 h), the cell number almost halved (−47.7%): fewer new cells migrated into the channel and fewer met the inclusion criteria. This trend continued, the absolute cell count decreased successively and reached the minimum after 150 h. This corresponds to a 73.8% decrease when comparing cell numbers at ex vivo times of 72 h and 150 h.

### 2.2. Analysis of Fluorescence Staining of Neutrophils

#### 2.2.1. Annexin V and PI Staining of Neutrophils

**Figure 3:**
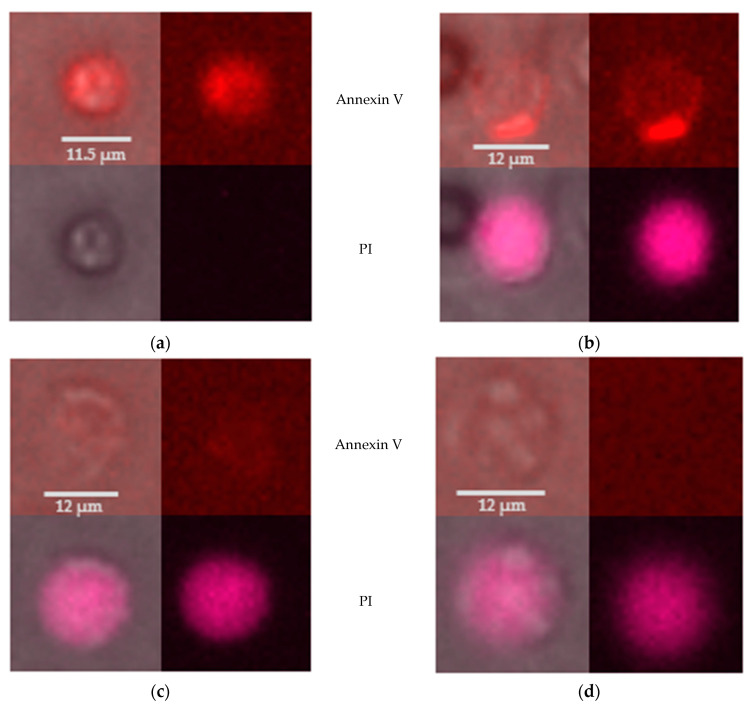
PMNs 72 h post withdrawal at different stages of the apoptotic process; left images: phase contrast + fluorescence channel; right images: fluorescence channel only; annexin V-FITC staining (top row), PI staining (bottom row). (**a**) Early apoptotic PMN: annexin V^+^ but PI^−^ (**b**) Apoptotic PMN: annexin V^+^ and PI^+^ (**c**) Late apoptotic PMN: weakly annexin V^+^ and PI^+^ (**d**) Late apoptotic/necrotic PMN: annexin V^−^ but PI^+^.

Analysis of fluorescent staining of PMNs showed that annexin V^+^ and/or PI^+^ cells were present after several hours of observation. Annexin V^+^ and PI^−^ cells are regarded to be in an early apoptotic process (Figure 3a). PMNs that were stained simultaneously by annexin V and PI are categorized as (late) apoptotic (Figure 3b,c). Annexin V^−^, but PI^+^ cells are described as late apoptotic/necrotic (Figure 3d).

To investigate the average percentage of PMNs stained by annexin V-FITC or PI after several hours ex vivo, phase contrast images were first used to determine the total number of PMNs in the channel at a given time point. In the next step, the number of fluorescent signals was identified and the percentage of fluorescent cells in the total number of cells was calculated. 

**Figure 4:**
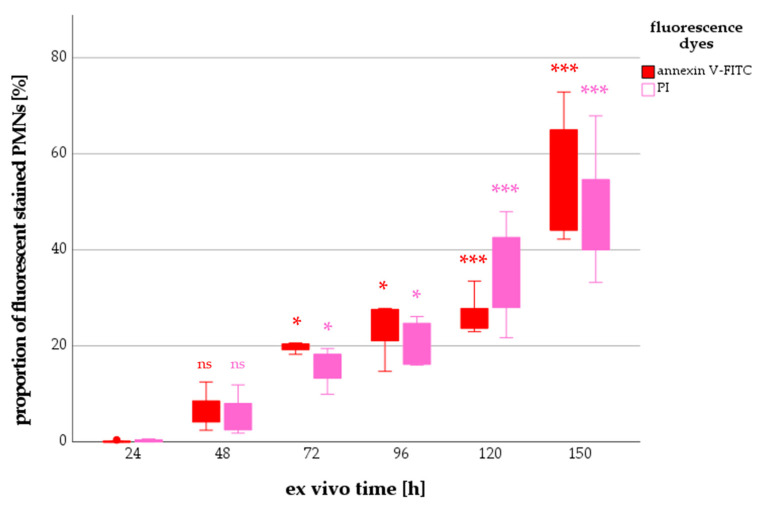
Number of non-surviving PMNs with increasing ex vivo time. Significant differences between groups compared with an ex vivo time of 24 h are red or pink colored (ns = not significant; * *p* < 0.05; *** *p* < 0.001).

There were hardly any annexin V^+^ nor PI^+^ PMNs 24 h post withdrawal. The median percentage of PMNs stained by annexin V-FITC increased over time from 7.2% (48 h) to a maximum of 61.2% (150 h). A similar trend was seen for PI^+^ cells: the median percentage increased from 7.0% (48 h) to 44.2% (150 h) (Figure 4). 

#### 2.2.2. Comparison of DAPI-Stained PMNs from Different Isolation Methods

**Figure 5:**
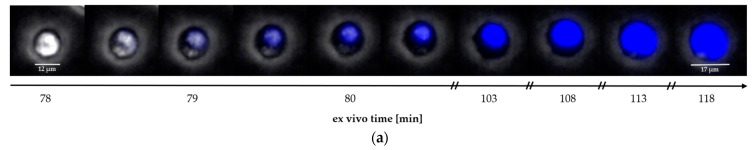

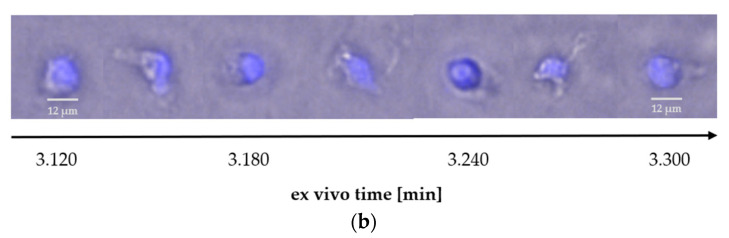
Examples of DAPI-stained PMNs from different isolation methods. (**a**) DAPI staining in a non-migrating PMN isolated by DGS. The fluorescence signal increased strongly in intensity within 30 min. The cell diameter was initially 12 µm (ex vivo time: 78 min) and increased to 17 µm (118 min). (**b**) DAPI staining in a vital PMN after isolation without centrifugation using gelafundin as sedimentation enhancer. The intensity of the fluorescence signal in the migrating PMN did not increase significantly over time. The diameter of the PMN remained constant over time at 12 µm.

NETosis was observed within 8 h when PMNs were isolated by density gradient separation [17,27,28]. The DAPI staining was seen within minutes and only in non-migrating cells, due to NETotic processes. The cell diameter of a representative PMN increased from 12 µm at the beginning to ultimately 17 µm within 40 min (Figure 5a). 

In contrast, we saw intracellular DAPI signals in vital, migrating cells without a sudden beginning, with low intensities slowly increasing with time, due to diffusion through the intact plasma membrane. The cell diameter remained constant (12 µm) (Figure 5b). 

#### 2.2.3. Progress of Median ROS Intensity

Living neutrophils can produce reactive oxygen species (ROS). The mean of the median fluorescence intensity [artificial fluorescence units; afu] remained almost constant along the ex vivo time (Table 2). After initial ROS production (up to 24 h), the formation hardly increased over the further observation period. The existing rhodamine could also be eliminated (thus losing intensity) and the new formation compensates for the loss. 

## 3. Discussion

Koo et al. analyzed granulocyte concentrates (GC) after apheresis with respect to neutrophil migration and viability [29]. In comparison, our data showed a migration length of neutrophils that was twice as long. Furthermore, the percentage of apoptotic PMNs was comparable (24 h), but the number of dead cells in GC was higher (24 h). After 48 h, GC had a lower percentage of apoptotic cells but a higher percentage of dead cells. However, these results of Koo et al. [29] were obtained by flow cytometry (Table 3).

Consequently, *g*-forces acting on granulocytes during apheresis may lead to a reduction in both migratory activity and lifespan. These results are consistent with the recently published work of Hundhammer et al. demonstrating a paralytic effect of centrifugation on granulocyte functionality as a function of applied *g*-time [26].

Our results lead to the conclusion that blood PMNs in vitro—with a lifespan up to 150 h (6.25 d)—are not that short-lived cells. The expected lifespan of non-activated neutrophils is much longer than previously thought. The results of Pillay et al. take a similar view: ^2^H_2_O-labelled neutrophils revealed an median lifespan of 5.4 days in vivo [8]. In those GC analyzed by Koo et al., more than 80% of the cells were still alive after 2 days ex vivo [29]. Moreover, of interest and consistent with the hypothesis is the work of Keating et al. which has shown that by day 5 of storage of non-leukoreduced red blood concentrates a mean percentage of 24.3% of all neutrophils are stained by annexin V (=being apoptotic) [30]. Comparing our neutrophil lifespan with the results of Patel et al. on monocytes, we obtained similar results at least for the intermediate and nonclassical cells [31] (Table 4). 

Reasons for the contrasting results—compared with the previously assumed maximum neutrophil circulation half-time in peripheral blood of less than 24 h [6,7]—could be that in previous studies (which used ex vivo manipulations, e.g., centrifugation), the circulating half-life of neutrophils was underestimated due to cell activation and homing [8]. Furthermore, neutrophils could become caught in the marginated pool. In addition, there are subtypes of neutrophils that are associated with a longer lifespan [32]. Further research on this issue is required, as definitive clarification has not yet been achieved.

Neutrophils are said to be able to die in various ways. The possible modes of death have been the subject of a variety of publications to date. Some papers describe up to seven possible modes, others less [11,12]. Nonetheless, apoptosis and NETosis undeniably play an important role [4,5,13]. 

Detection of apoptosis has been frequently performed using annexin V and PI in flow cytometric analyses [21,22,23]. In this static observation, cells were classified into four categories based on their fluorescence signals (Table 5) [19,20,33]: 

In contrast, we used live-cell fluorescence microscopy for apoptosis detection. The biggest advantage of this method is the dynamic observation which means seeing a progression of fluorescence signals. There are annexin V^+^ cells, which later become PI^+^ and in further observation, the annexin V signal fades away. Using only flow cytometry, one would classify this specific neutrophil first as apoptotic, then as late apoptotic or necrotic, and finally as dead. To avoid discrepancies—static vs. dynamic observation—we applied a slightly modified classification for fluorescence microscopy (Table 6): 

Annexin V^+^ PMNs were thought to be in a stage of apoptosis. The morphological cell changes were consistent with this assumption. However, annexin V is not a specific apoptosis marker. A positive fluorescence signal has also been described in necroptosis or necrosis for example [33,34]. In our study, we have not yet performed trials with other apoptosis markers. The joint detection of phosphatidylserine and calreticulin could be used as more informative detection of apoptotic cells in future studies [35].

Our results suggest that neutrophils isolated by DGS die in a different manner than those obtained without centrifugation steps. We saw that DAPI staining is not automatically associated with NETosis. NETosis is supported by a fast-appearing DAPI signal that increases in area and shows increasing intensity. DAPI staining of PMNs isolated by DGS showed these features [15,16,17]. In contrast, DAPI staining of native granulocytes showed entirely different characteristics: slow appearance, long-lasting, comparable intensity over time, migrating cells. These cells were not NETotic, but, in our opinion, most likely apoptotic. 

Limitations: The current experimental setup differed from those previously published [15,17]. Basic differences were the use of other µ-slides (µ-Slides VI 0.1 vs. 3D-µ-Slide), the PMNs must actively migrate from the reservoirs into the observation channel, no fetal calf serum in the reservoirs and a higher fMLP concentration (10 µM vs. 10 nM). Nevertheless, we do not think that the modified experimental setup is responsible for the strongly different results, but, essentially, the different isolation methods: DGS vs. sedimentation enhanced isolation with gelafundin. However, we cannot entirely rule out an interference.

For fluorescence analyses, we could only use two dyes per channel. Annexin V-FITC and DHR-123 as well as DAPI and PI could not be used simultaneously in one channel due to overlapping absorption spectra. 

Furthermore, the number of live cell imaging experiments is limited: n = 4 for migration analyses; n = 6 for annexin V and PI staining; n = 4 for DAPI staining and ROS intensity. Since these produced clear results, we considered the number of experiments to be sufficient. 

## 4. Materials and Methods

### 4.1. Study Plan

**Figure 6:**
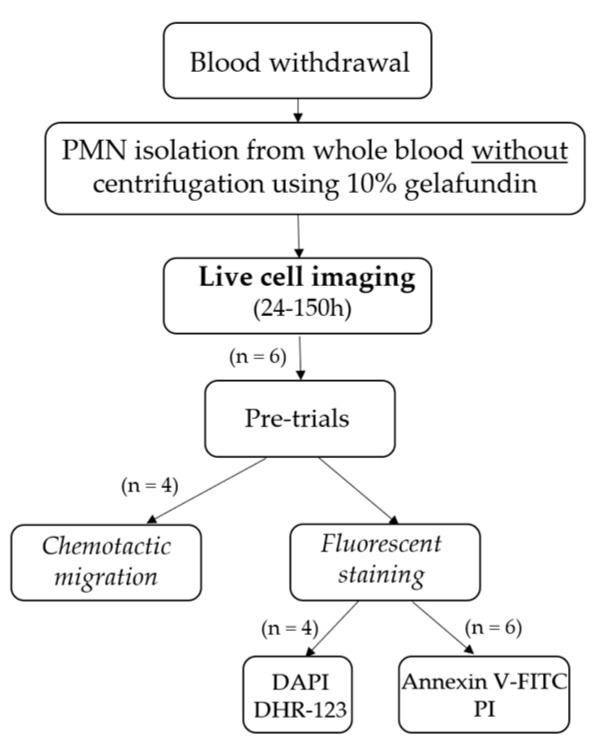
Schematic presentation of the used methods. On the one side, fluorescence analysis is performed using 4′,6-diamidino-2-phenylindole (DAPI) and dihydrorhodamine 123 (DHR-123) and on the other side, using annexin V-FITC and propidium iodide (PI).

To address the question of whether non-centrifuged PMNs are indeed short-lived cells and how they meet their demise, live-cell imaging was employed (Figure 6). 

### 4.2. Blood Withdrawal and PMN Isolation

The in vitro experiments were approved by the Ethics Committee (No. 20-1919-101) of the University of Regensburg (Regensburg, Germany. The donors were clinically recognized as healthy and were not taking any medications. After the donors gave their informed consent, peripheral venous blood was withdrawn. A lithium heparin-anticoagulated blood collection tube and one serum clot activator tube were used for this purpose (all blood collection materials were from SARSTEDT AG & Co. KG, Nuembrecht, Germany).

PMNs were isolated without centrifugation. Therefore, 10% gelafundin^®^ ISO (40 mg/mL; B. Braun SE, Melsungen, Germany) was added to whole blood. The supernatant containing most native PMNs was collected after 30 min.

The serum monovettes were centrifuged for 10 min at 1.181× *g* and room temperature (Heraeus™ Megafuge™ type 1.0 R, Thermo Fisher Scientific, Waltham, MA, USA). Subsequently, the supernatant, which contained blood serum only, was extracted and further used. 

### 4.3. Experimental Setup

**Figure 7:**
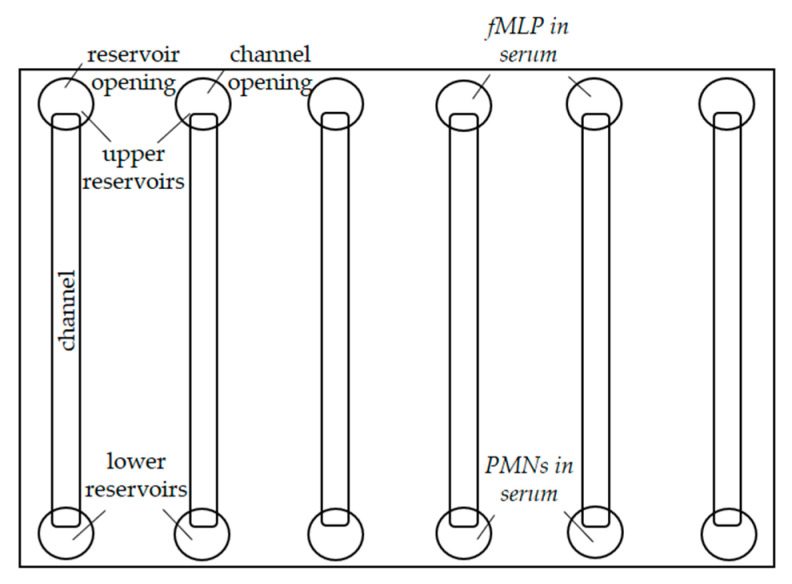
Schematic representation of the µ-Slides VI 0.1 used.

µ-Slides VI 0.1 (Ibidi GmbH, Graefelfing, Germany) were used to study the migration and fluorescent staining of PMNs. A slide consists of six channels, with a reservoir attached to the top and bottom of each channel (Figure 7). 

After filling the channels, the slide was incubated for 45 min under constant humid conditions at 37 °C and 5% CO_2_. Subsequently, the reservoirs were filled.

N-Formylmethionine-leucyl-phenylalanine (fMLP; Sigma Aldrich, St. Louis, MO, USA) was used as a chemoattractant. The oxidation of dihydrorhodamine 123 (Thermo Fisher Scientific, Waltham, MA, USA) into rhodamine 123 was used as an indicator of intracellular ROS production. Free (fast, widespread and bright glowing) and intracellular (slow, bruising and dimly glowing) DNA was stained with DAPI (Sigma Aldrich, St. Louis, MO, USA). To show loss of plasma membrane asymmetry annexin V-FITC (BioLegend, San Diego, CA, USA), and to detect nonviable cells PI (Thermo Fisher Scientific, Waltham, MA, USA) were added. The composition of the channel and reservoir fillings are listed below (Table 7). 

Live cell imaging was performed with a DMi8 microscope, Leica DFC9000 GT camera, Leica Application Suite X software (version 3.4.2.18368; all supplies from Leica Microsystems GmbH, Wetzlar, Germany), a top stage incubator (Ibidi GmbH Graefelfing, Germany) and a pE-4000 LED light source (CoolLED, Andover, UK). Phase contrast images and two fluorescence images per cycle were automatically recorded. The recording frequency varied and was either 30 s for migration analysis or 10/30 min for fluorescence analysis. The observation period was at least 24 h and a maximum of 150 h. 

### 4.4. Data Analysis

IMARIS 9.02 (Bitplane, Zurich, Switzerland), Excel (Microsoft Corp., Redmond, WA, USA) and IBM^®^ SPSS^®^ (version 28.0.0.0, IBM Corp., Armonk, NY, USA) were used for data analysis. Before examining statistical differences between the groups, statistical outliers were excluded. For statistical analyzation normal distribution tests (Kolmogorov–Smirnov) were performed. Since, in all cases, the data did not show normal distribution, comparison of multiple groups (Kruskal–Wallis tests) were performed followed by post hoc tests (Bonferroni). *p* values below 0.05 were considered significant if not stated otherwise. Small circles in graphics mark statistical outliers (≥1.5 × IQR) and asterisks display extreme values (≥3 × IQR). 

#### 4.4.1. Migration Analysis

PMNs in autologous serum migrated towards an fMLP gradient from the lower reservoirs into the collagen matrix of the channels and were observed there.

Migration activity was compared during 15 min observation periods (recording frequency: 30 s) after ex vivo periods from 24 h to 150 h. We investigated the following parameters (Table 8): 

#### 4.4.2. Fluorescent Staining

Fluorescent staining of PMNs was examined along the ex vivo time. Briefly, percentage medians of all PMNs in the observable part of the channels stained by annexin V or PI after 24 h, 48 h, 72 h, 96 h, 120 h and 150 h were investigated. 

In addition, the characteristics of DAPI-stained PMNs from different isolation methods were visually compared: density gradient separation vs. sedimentation enhanced isolation without centrifugation. 

To examine the progress of ROS intensity the median intensities after 24–150 h were investigated. Afterwards the mean median intensities were compared. 

## 5. Conclusions

Isolation steps with centrifugation significantly impair the functions of neutrophils. Native PMNs are not at all immediately death prone cells; instead, a considerable amount showed a relevant migratory activity even after more than six days post withdrawal. The cells become consecutively apoptotic rather than NETotic after many hours ex vivo. For further studies on neutrophils, it is crucial to minimize *g*-time load.

In conclusion, the behavior of most native neutrophils—long migratory activity as well as late annexin V and PI staining—is different from PMNs isolated by density gradient separation. Therefore, we claim that NETosis after DGS is the consequence of applied *g*-forces and not the physiological mode of death of neutrophils. 

## Figures and Tables

**Table 1:** Characteristics of the study population.

Characteristics	Values (Range)
Number of Subjects	6
Gender (female/male)	4/2
Median Age (years)	21 (20–58)

**Table 2:** Progress of median ROS intensity along the ex vivo time.

Ex Vivo Time [h]	Mean of Median ROS Intensity [afu]
24	13.8 ± 1.9
48	13.1 ± 1.0
72	14.2 ± 3.2
96	14.1 ± 2.1
120	12.9 ± 0.8
150	13.8 ± 0.9

**Table 3:** Comparison of TL and the percentage of apoptotic/dead PMNs in GC [29] as well as after <1 kgs isolation after 24 h and 48 h, respectively.

Samples	Time [h]	TL [µm] (15 min)	TL [µm] (30 min)	Apoptotic [%]	Necrotic/Dead [%]
GC	24	57 (calculated)	114	0.42	9.84
<1 k*g*s	24	126	252 (calculated)	<0.5	<0.5
GC	48	31.7 (calculated)	63.5	0.79	17.4
<1 k*g*s	48	63	126 (calculated)	7.2	7.0

**Table 4:** Lifespan of human blood monocytes in vivo [31].

Cell Type (Human)	Mean Lifespan (Days)	Test Method
Monocytes (classical)	1.0 ± 0.26	deuterium-labeled glucose
Monocytes (intermediate)	4.3 ± 0.36	deuterium-labeled glucose
Monocytes (nonclassical)	7.4 ± 0.53	deuterium-labeled glucose

**Table 5:** Characteristics of fluorescence signals for apoptosis classification of PMNs using flow cytometry.

Cell State	Annexin V	PI
Vital	−	−
Apoptotic	+	−
Late apoptotic/necroptotic/necrotic	+	+
Dead	−	+

**Table 6:** Modified characteristics of fluorescence signals for apoptosis classification of PMNs using fluorescence microscopy.

Cell State	Annexin V	PI
Vital	−	−
Early apoptotic	+	−
Apoptotic	+	+
Late apoptotic	+ (weak)	+
Late apoptotic/necrotic	−	+

**Table 7:** Composition of a channel and its reservoir fillings of the µ-Slides VI 0.1 used.

Upper Reservoir (60 µL)	Channel (5 µL)		Lower Reservoir (60 µL)
fMLP [10 µM] in autologous serum	Cell medium (33%) DAPI [2 µg/mL] DHR-123 [6.6 µM] Collagen type I (50%)	Cell medium (38%) Annexin V-FITC [7.6 µg/mL] PI [4.6 µg/mL] Collagen type I (50%)	PMNs supernatant and autologous serum (each 50%)

**Table 8:** Analyzed parameters as indicators of the migration properties of PMNs, defined according to [28].

Parameter (Abbreviation)	Unit	Description
Track length (TL)	[µm]	Total track of a PMN within 15 min
Mean track speed (TS)	[µm/s]	Migration speed of the tracked PMN
Number of PMNs	1	Total number of analyzed PMNs meeting inclusion criteria: TL ≥ 25 µm + track duration ≥ 450 s

## Data Availability

The data presented in this work are available on request from the corresponding author.
